# The effect of synthetic bone graft substitutes on bone formation in rabbit calvarial defects

**DOI:** 10.1007/s10856-020-06483-6

**Published:** 2021-01-21

**Authors:** Nikola Saulacic, Masako Fujioka-Kobayashi, Yasushi Kimura, Ava Insa Bracher, Claudio Zihlmann, Niklaus P. Lang

**Affiliations:** 1 Department of Cranio-Maxillofacial Surgery, Faculty of Medicine, Inselspital, Bern University Hospital, University of Bern, Bern, Switzerland; 2 Department of Oral and Maxillofacial Surgery, National Defense Medical College Hospital, Saitama, Japan; 3 Geistlich Pharma AG, Wolhusen, Switzerland

## Abstract

The aim of this study was to evaluate the influence of the intensity of the biomimetic hydroxyapatite (HA) coating of α-tricalcium phosphate (α-TCP) on biomaterial degradation and bone formation. Twenty-four female NZW rabbits of approximately 12 weeks of age were used. Critical size defects were randomly treated with 3%:97% HA:α-TCP (BBCP1), 12%:88% HA:α-TCP (BBCP2), and 23%:77% HA:α-TCP (BBCP3), respectively or sham. All defects were covered with a resorbable collagen membrane. Animals were euthanized after 3 and 12 weeks of healing and samples were investigated by micro-CT and histologic analysis. Ingrowth of newly formed woven bone from the original bone at 3-week healing period was observed in all samples. At the 12-week healing period, the new bone in the peripheral area was mainly lamellar and in the central region composed of both woven and lamellar bone. New bony tissue was found on the surface of all three types of granules and at the interior of the BBCP1 granules. Samples with 3% HA showed significantly less residual biomaterial in comparison to the other two groups. Furthermore, BBCP1 significantly promoted new bone area as compared to other three groups and more bone volume as compared to the control. Within its limitations, this study indicated the highest degradation rate in case of BBCP1 concomitant with the highest rate of bone formation. Hence, formation of new bone can be affected by the level of biomimetic HA coating of α-TCP.

## Introduction

Autogenous bone grafts and bone graft substitutes have been frequently used for the treatment for atrophic jaw bone in implant dentistry. The advantage of autogenic bone grafts is its osteoconductivity and possibly osteoinductivity [1]. Nevertheless, granulated bone substitutes were applied to avoid the necessity of two surgical sites with donor site morbidity [2]. The graft has to possess appropriate characteristics to allow three-dimensional stability in relation to the type of defect [3]. In fact, granulated bone grafts result in highly predictable treatment outcomes when applied together with membranes following the principle of guided bone regeneration [4].

Bone graft substitutes have to be biocompatible and osteoconductive, but healing times may be prolonged when compared to the use of particulated autografts. Ideally, degradation of bone graft substitute materials should correspond to the degree of new bone formation. In the posterior region of the mandible, the percent of residual allograft may be up to 69% and of newly formed bone less than 5% after 6 months of healing [5]. Deproteinized bovine bone mineral is a widely applied xenogenic bone graft that is without a risk for an immune response. In comparison to autografts, this xenogenic bone graft shows a slower and incomplete resorption [6, 7]. As an alternative to the xenogenic materials a variety of alloplastic biomaterials such as calcium phosphates, calcium sulfate, bioactive glasses, and hard tissue replacement polymeric [8] were developed with different physico-chemical properties. Calcium phosphates, in particular hydroxyapatite (HA) and tricalcium phosphate (TCP) have been in the focus of biomaterial research for bone augmentation procedures due to their composition resembling natural mineral bone. An osteoconductive potential for calcium phosphates has been confirmed. Calcium phosphates, however, differ in their level of degradation rate with HA being the least degradable when compared to TCPs. Furthermore, TCPs are divided into α- and β-TCP that also display distinct degradation pattern [9]. Fast degradation of β-TCP scaffolds resulted, however, in unsatisfactory bone formation [10, 11]. α-TCP yielded even faster biodegradability [12] due to the differences in structure, density, and solubility [13], and because of this rapid biodegradability its clinical applications is practically limited to α-TCP bone cements [13].

Biphasic synthetic calcium phosphates (BCP) that comprise different ratios of HA and TCP were developed in an attempt to combine the desired properties of degradation and osteoconduction [14, 15]. HA is an inert material, which is biologically relatively undegradable, and, as such, is the limiting factor for the degradation kinetics of BCP [16]. It has thus a good space-maintaining capacity, whereas TCP is typically rapidly degraded and replaced with new bone. Consequently, BCPs of the HA/β-TCP type have been developed differing in the ratio of HA to β-TCP with the goal to fine-tune the degradation in vivo. Unexpectedly, graft volumes, number of osteoclasts, and blood vessels for different ratios of HA/β-TCP were similar [17–20]. Furthermore, a substantial bone formation was observed during early healing periods using the HA/α-TCP in a block form [21, 22] or as a putty embedded in porcine collagen [23]. The biomaterial, however, did not undergo marked degradation during bone tissue modeling and remodeling and remained as a stable scaffold even on a long term [24].

This study was designed to evaluate the influence of the level of the biomimetic HA coating of α-TCP in particulate form. The hypothesis was that the lower HA content might lead to the higher rate of material degradation concomitant with the higher rate of new bone formation. Hence, the effects on bone formation of three prototypes displaying different biomimetic HA concentrations were assessed on bone tissue modeling and remodeling during healing of critical size defects in rabbits.

## Materials and methods

### Materials

**Fig. 1:**
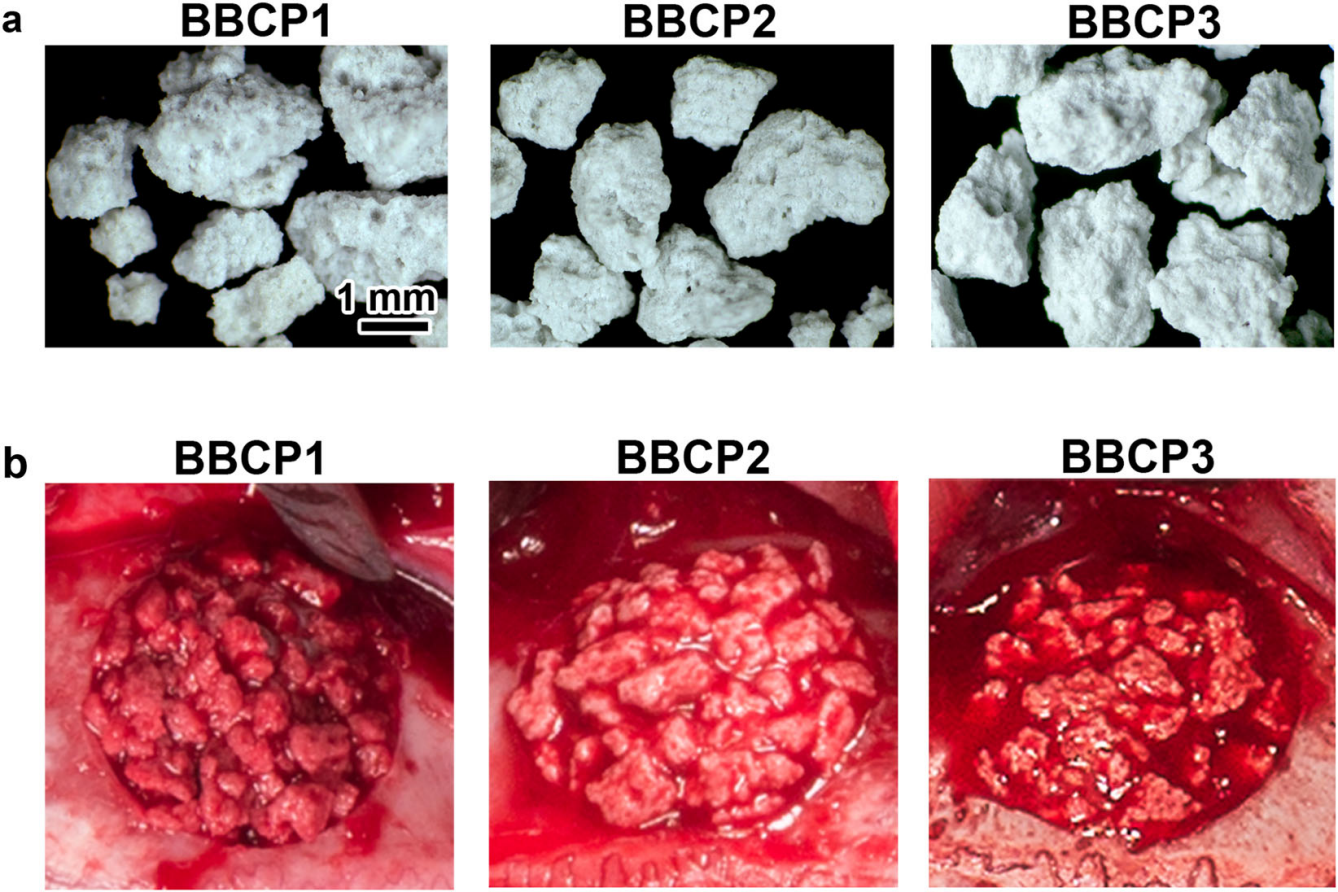
**a** Surface appearance of the particles of BBCP1, BBCP2, and BBCP3. The rough surfaces with pores were observed on each particle. **b** The images of the bone defects in rabbit parietal bone during surgeries. The materials were implanted in each defect, followed by mixing with autologous blood collected from auricular artery

The HA/α-TCP composite materials were kindly provided by Geistlich Pharma AG (Wolhusen, Switzerland). HA/α-TCP is a composite bilayered material composed of an α-TCP core epitactically coated with a biomimetic nanocrystalline HA layer of a defined thickness (bilayered biphasic calcium phosphate (BBCP)). The sintered core material is obtained by mixing powders of calcium and phosphate donors in appropriate ratios, and sintering the mixture at high temperature. The coating is obtained through the transition of the surface of the sintered TCP core material into biomimetic HA. The biomimetic HA coating of BCP has a crystal size of 18 × 8 x 38 nm, similar to that of natural bone mineral. The following prototypes were produced 3%:97% HA:α-TCP (BBCP1), 12%:88% HA:α-TCP (BBCP2), and 23%:77% HA:α-TCP (BBCP3). All BBCP prototypes were provided in particulate form. The size of all tested particles ranged from 0.7 to 2.0 mm (Fig. 1a). The surface of all particles presented numerous macro-pores. Each bone substitute was separately packed for each defect and sterilized before surgery.

### Animals

In total, 24 adult female New Zealand white rabbits (3.0–4.0 kg) were purchased from Charles Rivers Laboratories (Romans, France) and subject to calvarial defect assays. The rabbits were housed 2 weeks prior to surgery for the acclimatization and throughout the whole experiment in the Central Animal Care Facility at the University of Berne with an adjusted climate (temperature 19–21 °C, humidity 45% ± 10%, a light:dark cycle of 12:12 h), without excessive or disturbing noises. The animals were fed a standard diet and given water ad libitum. The study was designed as a prospective, randomized study following the ARRIVE guidelines for preclinical in vivo studies (NC3Rs, UK), approved by the Committee for Animal Research, State of Berne, Switzerland (BE 89/17).

### Anesthesia and surgery

Rabbits were premedicated with ketamine 65 mg/kg s.c (Narketan^®^, Vetoquinol AG, Bern, Switzerland), xylazine 4 mg/kg (Xylapan^®^, Vetoquinol AG, Bern, Switzerland), and buprenorphine 0.03 mg/kg (Temgesic^®^, Reckitt Benckiser, Wallisellen, Switzerland) in the neck area mixed in the same syringe and left undisturbed for 10–15 min. After reaching the appropriate depth of sedation, the eyes were lubricated (Bepanthen Augen und Nasensalbe, Bayer Vital GmbH, Leverkusen, Germany), and pure oxygen administered by means of a facemask. A 22 G IV catheter (Vasofix^®^ Safety, B. Braun Melsungen AG, Sempach, Switzerland) was inserted in the auricular vein. A size R3 or R4 V-Gel^®^ (Docsinnovent Ltd, London, UK) was inserted and the anesthesia maintained with 0–1% isoflurane (Forene, Abbvie AG, Baar, Switzerland) vaporized in pure oxygen through a Jackson Rees modified T-piece breathing system. Rabbits were spontaneously ventilating throughout the procedure. Monitoring included clinical assessment of the anesthetic depth by a pulse oximetry, capnography, gas analysis, and rectal temperature (Datex-Ohmeda S3, Helsinki, Finland). Clinical variables were recorded at 5 min intervals. Meloxicam 0.3 mg/kg s.c. (Metacam^®^, Boehringer Ingelheim, Ingelheim, Germany) and penicillin 150,000 IU/mL + benzathine penicillin 150,000 IU/mL s.c. (Duplocillin^®^, MSD Animal Health, Luzern, Switzerland) 0.01 mL/kg were administered before surgery. NaCl 0.9% (B. Braun Medical AG) was administered at 5 mL/kg/h throughout the procedure.

The surgical area was shaved and disinfected. A local anesthesia was introduced with 0.5 mL of 1% lidocaine HCl (Lidocaïne, Bichsel, Interlaken, Switzerland). A 3.5-cm incision was made from the nasal bone to the mid-sagittal crest. The parietal bone was exposed following the elevation of the periosteum, and two 10-mm diameter bone defects were prepared with a trephine and diamond round burs under copious irrigation with sterile saline. Maximal care was taken to avoid injury of the dura mater.

The following four treatment modalities were randomly allocated: (i) sham, (ii) BBCP1, (iii) BBCP2, and (iv) BBCP3. The weight of all tested materials was about 0.15 g per defect. Allocation of the applied treatment modalities was randomized according to the systematic random protocol (www.randomization.com). The 600 µL of blood was collected from auricular artery per an animal. The 300 µL of blood was used to mix with each granule and implanted into a defect and the 300 µL filled up into the negative control (sham) (Fig. 1b). After implantation of the materials, the 12.5 mm × 13.0 mm-sized resorbable collagen membrane (Geistlich Bio-Gide^®^, Geistlich Pharma AG, Wolhusen LU, Switzerland) was used to cover the defect site. The periosteum and skin were closed with interrupted sutures in layers using 4–0 Vicryl^®^ and 4–0 Monocryl^®^ sutures (Ethicon, Somerville, NJ, USA). The wound surface was sealed with a spray film dressing (OPSITE^®^ SPRAY, Smith & Nephew, London, UK).

Once the surgery was completed, the rabbits were left to recover under infrared lights and administration of oxygen. Temperature, pulse rate, and respiratory rate were regularly monitored. When fully recovered, the rabbits were returned to the animal facility. Postoperative analgesia included buprenorphine 0.03 mg/kg s.c. every 8 h for 2–3 days and meloxicam 0.3 mg/kg once daily for 5 days. Pain was assessed at regular intervals (composite pain scale and grimace scale) and rescue analgesia was administered if necessary. Water and food consumption, fecal and urine production as well as postoperative weight were monitored.

### Euthanasia

Following the premedication with ketamine 65 mg/kg and xylazine 4 mg/kg s.c. in the neck area, Esconacron 120 mg/kg i.v. (Streuli Pharma AG, Uznach, Switzerland) was injected for the euthanasia.

### Micro-CT analysis

The defect sites were removed and fixed in 10% neutral formalin for 7 days at room temperature, then replaced in 70% ethanol at 4 °C, followed by PBS rinsing. Thereafter, the specimens were subjected to micro-CT scans using a desktop Cone-Beam scanner (micro-CT 40, Scanco Medical AG, Brüttisellen, Switzerland). The X-ray source was set at 70 kV with 114 μA at an isotropic voxel size of 18 µm, which showed an image matrix of 2048 × 2048 pixels. The micro-CT images were then analyzed and reconstructed by using 3D structural analysis software (Amira, Visualization Sciences Group, Düsseldorf, Germany). The volume of interest was selected corresponding to the dimensions of the defect sites, with a diameter of 10-mm full-thickness cylinders. Bone volume (BV, mm^3^), bone density (BD, relative % to initial bone at the defect site), residual material volume (RMV, mm^3^), and mineralized tissue volume (MTV, mm^3^; BV + RMV) were calculated after the segmentation.

### Histology and histomorphometry

**Fig. 2:**
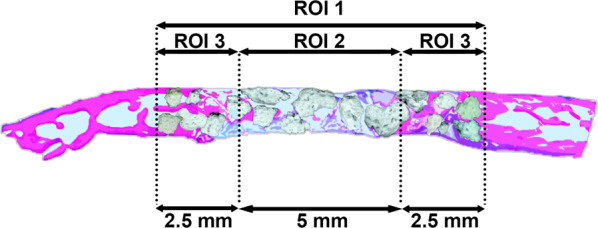
ROIs for histomorphometry. ROI 1 (whole defect area), ROI 2 (middle area in a defect), and ROI 3 (lateral area in a defect) were investigated for each parameter

Histology was performed on all prototypes mixed with blood and on all defects. The specimens were trimmed, dehydrated in ascending concentrations of ethanol, and embedded in methylmethacrylate without decalcification. The embedded tissue blocks were cut in a sagittal direction at the middle of the defects into approximately 800-µm thick ground sections using a slow-speed diamond saw (VC-50; LECO, St. Joseph, MI, USA). After mounting on acrylic glass slabs, the sections were ground and polished to a final thickness of 200 µm (Knuth Rotor-3; Struers, Ballerup, Denmark). The labeled bone was digitally photographed under a fluorescent microscope (Nikon Eclipse E800; Nikon, Tokyo, Japan). The sections were stained with toluidine blue combined with fuchsin. The images were photographed under a Wild Heerbrugg M400 ZOOM Makroskop for overviews and a light microscope (Nikon Eclipse E800) equipped with a digital imaging system (NIS Elements; Nikon). Morphometric analysis was performed by a graphic software (Photoshop CS6; Adobe, San Jose, CA, USA) using the corresponding 10-mm initial defect area as the region of interest 1 (ROI 1). Furthermore, each ROI 1 was divided into ROI 2 (central area of a defect) and ROI 3 (peripheral area of a defect) (Fig. 2). The parameters including horizontal defect closure (%), new bone area (NBA, relative % to total augmentation area), bone marrow area (relative % to total augmentation area), residual material area (RMA, relative % to total augmentation area), mineralized tissue area (MTA, NBA + RMA; relative % to total augmentation area), and connective tissue area (CTA, relative % to total augmentation area) were calculated.

### Statistical analysis

For all the quantitative data, the means and all plots of values were represented. The statistical analysis was done by one-way analysis of variance with Tukey test by a statistical program (GraphPad Prism 7.0 software; GraphPad Software, Inc., La Jolla, CA, USA). The *p* values < 0.05 were considered significant.

## Results

Two rabbits had to be euthanized because of neurological symptoms or respiratory disorders within 1–2 days post surgery. Thus, 11 rabbits were sacrificed after healing periods of 3 weeks and 3 months each. By the time of sacrifice, there were no signs of wound dehiscence, exposures, inflammation, or infections at the surgical site. However, two defects in two animals had to be excluded from the analysis due to the damage of the dura. A total of 42 defects were analyzed, with *n* = 5 for the treatment modality and healing period and *n* = 6 for BBCP2 (3 weeks and 3 months).

### Micro-CT analysis

**Fig. 3:**
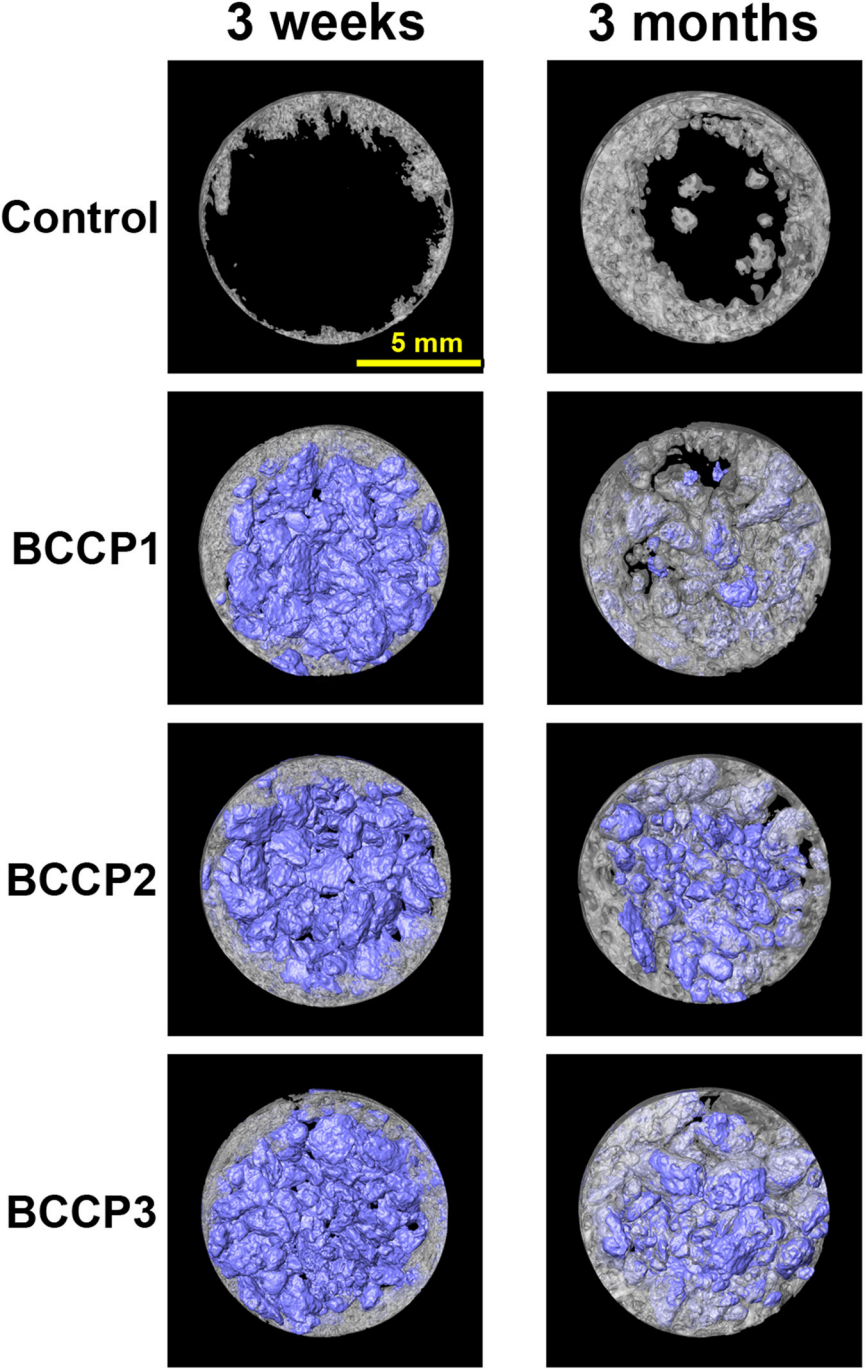
3D reconstructed micro-CT images of control (empty), BBCP1, BBCP2, and BBCP3 samples at 3 weeks and 3 months post surgery

**Fig. 4:**
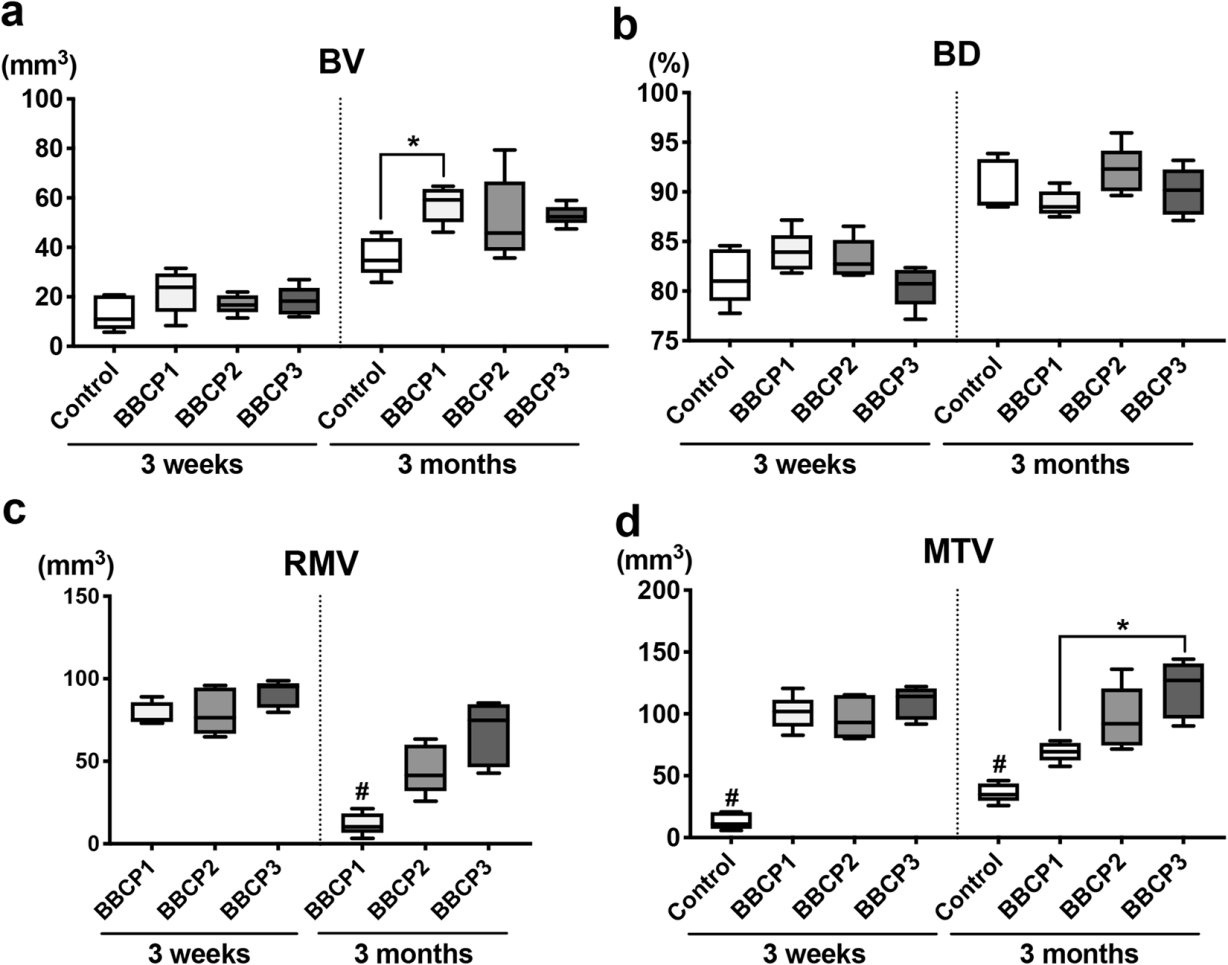
**a** Bone volume (BV, mm^3^), **b** bone density (BD, relative % to initial bone at the defect site), **c** residual material volume (RMV, mm^3^), and **d** mineralized tissue volume (MTV, mm^3^, BV + RMV) at 3 weeks and 3 months post surgery. As a reference, control bone samples were used to show initial bone volume in the defect and initial bone density. *denotes significant difference between the groups (*p* < 0.05). ^#^denotes significantly lower than any other modalities (*p* *<* 0.05)

At the 3-week timepoint, new bone formation as well as particulate distribution in the defects was assessed (Fig. 3). Ingrowth of new bone was observed at peripheral areas of the defect in all groups, and this infiltrated into the gaps between the granules. The quantitative analysis showed no significant differences in the BV or RMV between any group (Fig. 4).

At 3 months, most of the BBCP1 granules could not be identified anymore, whereas BBCP2 and BBCP3 granules were clearly visible. Accordingly, BBCP1 samples showed significantly lower RMV when compared with the other two BBCP groups (Fig. 4c). New bone was seen at the central area of the defects between the BBCP particles and to a lesser extent in the control group. Interestingly, BBCP1 group demonstrated significantly more BV when compared with the control group. The values for BD, however, did not differ significantly from each other (Fig. 4b). The MTV was related to the content of HA, with BBCP3 having significantly greater values than BBCP1 (Fig. 4d).

When compared longitudinally from 3 weeks to 3 months, both BV and BD in all tested groups significantly increased, while the RMV significantly decreased (data not shown). Moreover, MTV and MTD significantly decreased only in the BBCP1 group.

### Histological observation

#### Histology of granules after mixing with blood

**Fig. 5:**
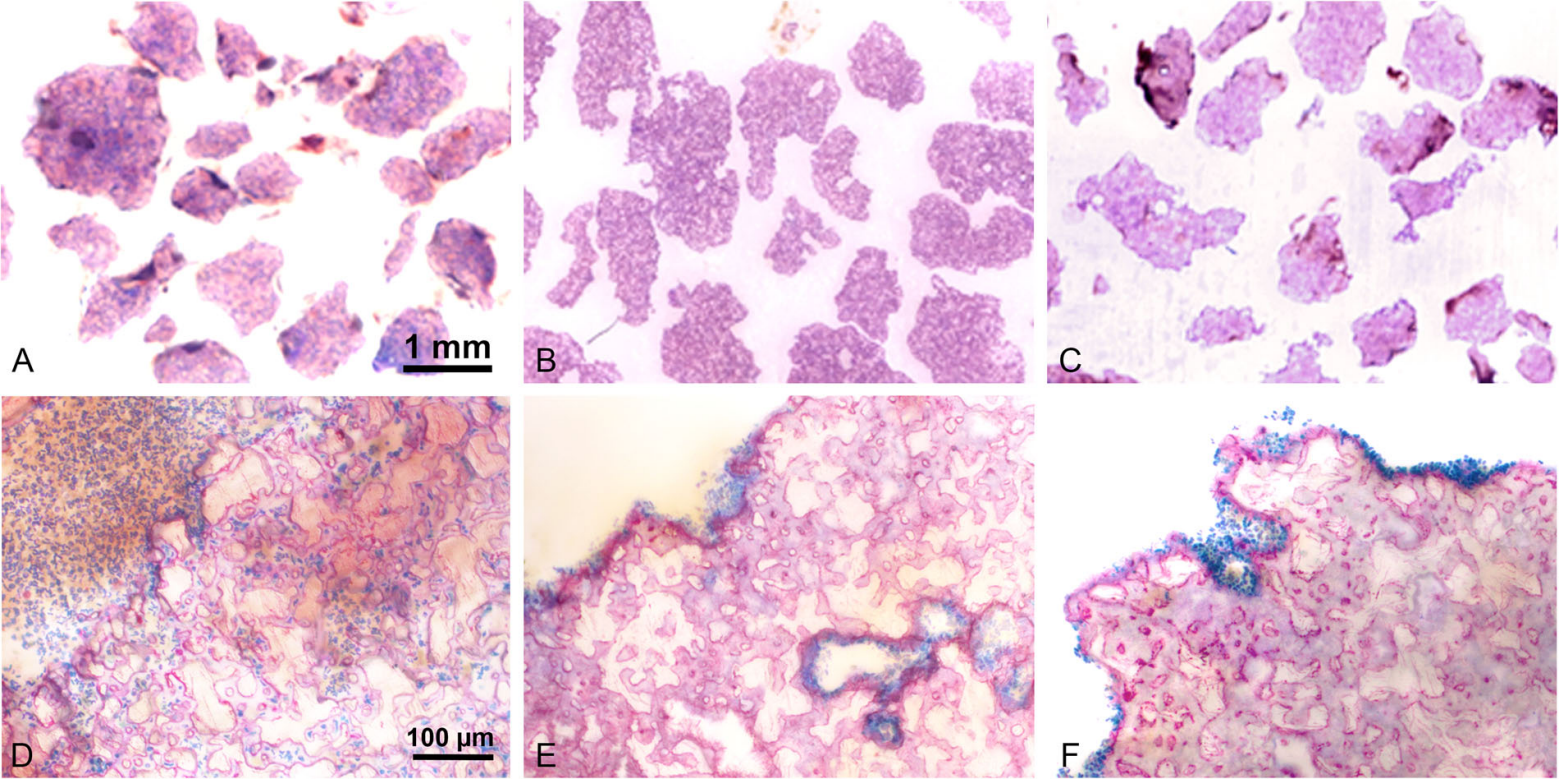
Low (above) and high (under) magnified sectional images of the BBCP1 (**a**, **d**), BBCP2 (**b**, **e**), and BBCP3 (**c**, **f**) after fixing with blood. Blood cells including red blood cells and leucocytes infiltrated clearly only into the pores of the BBCP1

Samples of granules after mixing with blood showed granules surrounded with a corona of blood cells (Fig. 5). White blood cells and erythrocytes were found to be present up to the center of each BBCP1 granule, the BBCP2 and BBCP3 granules were hardly infiltrated with cells.

#### Descriptive histology

**Fig. 6:**
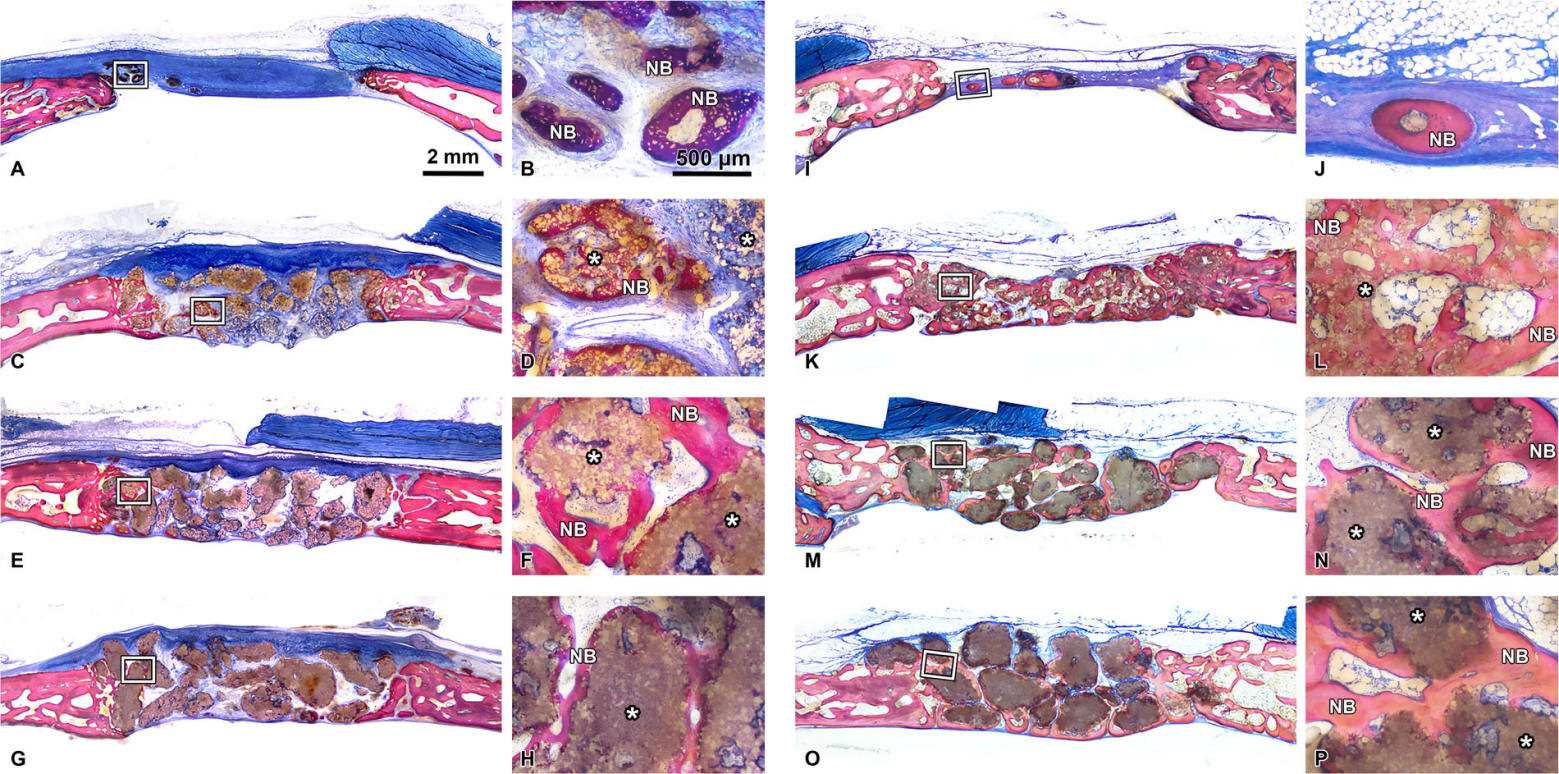
Representative histological images of control (**a**, **b**, **i**, **j**), BBCP1 (**c**, **d**, **k**, **l**), BBCP2 (**e**, **f**, **m**, **n**), and BBCP3 (**g**, **h**, **o**, **p**) groups at 3 weeks (left) and 3 months (right) of healing (toluidine blue and fuchsin staining). At 3 weeks, new bone is observed at the peripheral areas of the defect. The remnants of membranes are detectable covering the defect and the old bone. The outlined areas in the periphery of the defects (**a**, **c**, **e**, **g**) are magnified (**b**, **d**, **f**, **h**). All groups of granules (*) are showing contact to the newly formed woven bone (NB). At the 3 months of healing, new bone is seen in the central areas of the defect. The outlined areas (**i**, **k**, **m**, **o**) are magnified (**j**, **l**, **n**, **p**). Granules (*) are integrated in a newly formed lamellar bone (NB)

**Fig. 7:**
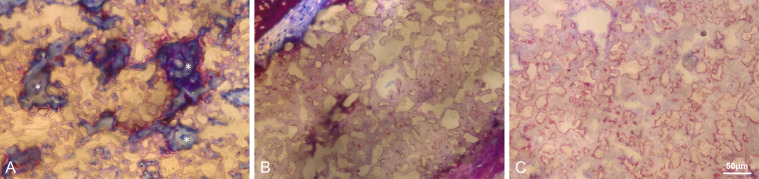
High magnification of the biomaterial at the center of defect after 3 weeks of healing. **a** At the interior of BBCP1 granules, multinucleated cells (*) and osteoblasts are identified inside the pores. **b** Direct contact of bone with BBCP2 granules is seen, with rare infiltration of the cells. **c** Small pores of the BBCP3 granules without cell infiltration

**Fig. 8:**
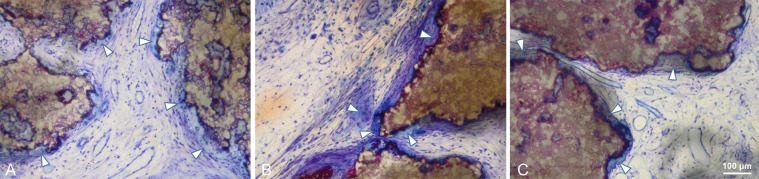
Center of the defect treated with BBCP1 (**a**), BBCP2 (**b**), and BBCP3 (**c**) granules after 3 weeks of healing. Granules are almost completely surrounded by a layer of multinuclear cells (arrowheads). Cell poor and highly vascularized loose connective tissue is observed between the granules

In general, no samples showed any signs of local non-physiological inflammation or infection (Fig. 6). The collagen membrane was still being present after 3 weeks. The newly formed woven bone was observed in the peripheral areas of the defects in all groups at 3 weeks by ingrowth from the edges of the pristine bone (Fig. 6). All test groups show good contact of the biomaterial to the new bone in the peripheral area. New bony tissue was also found in the BBCP1 granules’ interior (Fig. 7). In BBCP2 or BBCP3 samples, newly formed bone was restricted to large pores only and the outer surface. In the central areas of the defect, all BBCP prototypes were found embedded in a cell poor, but rich vascularized, connective tissue (Fig. 8). In areas of close proximity between granules, a condense sheath-like connective tissue with abundant multinucleated cells around the granules was observed. In the case of BBCP1, multinucleated cells were identified together with mesenchymal type of cells (presumably osteoblasts) throughout the small pores of the particles’ interior.

**Fig. 9:**
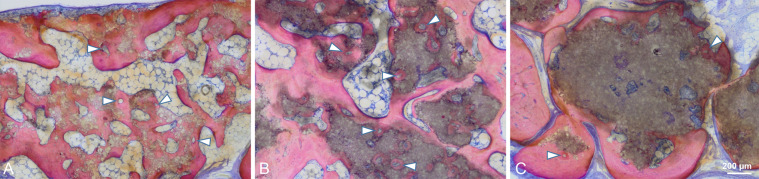
Periphery of defect treated with BBCP1 (**a**), BBCP2 (**b**), and BBCP3 (**c**) granules after 3 months of healing. Residual granules are embedded in the newly formed bone. Osteons (arrowheads) are observed within the newly formed lamellar bone and perforating the granules. The evidence for haematopoiesis with different degrees of cellularity is seen in all samples

**Fig. 10:**
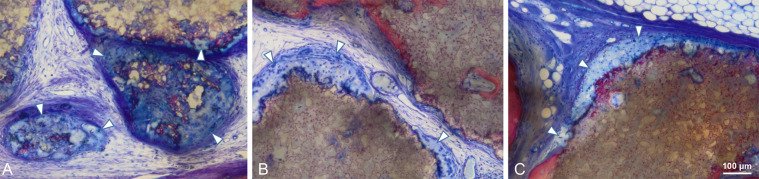
Center of the defects treated with BBCP1 (**a**), BBCP2 (**b**), and BBCP3 (**c**) granules after 3 months of healing. Non-integrated granules are covered with a plenty of multinucleated giant cells (arrowheads) and a condensed fibrous tissue

Newly formed bone at 3 months was observed in the central area in most of the sections of all groups, adjacent to dura mater rather than on the periosteal sides (Fig. 6). No remnants of the collagen membrane could be identified. Thin spikes of newly formed bone growing from the original bone were observed in control group, with a flattened area between the periosteum and dura in the central region (Fig. 6). The thickness of newly formed bone in BBCP groups was corresponding to the thickness of original bone, with a maintained space between the periosteum and the dura in the central region. The new bone in the peripheral area was mainly lamellar with structures resembling bone forming units (osteons). In the central region, new bone was composed of woven and lamellar bone. While the residual BBCP1 particles were observed localized in the center of the defects, the BBCP2 and BBCP3 granules were present throughout the whole defect. Residual BBCP1 particle fragments at the periphery of the defect were embedded in the newly formed bone (Fig. 9). Plenty of huge multinucleated cells were seen on the surface of the granules in all tested BBCP groups mostly in the center of the defect (Fig. 10). Corresponding to the 3 weeks observation period, a condensed fibrous tissue was found surrounding the BBCP particles.

In terms of the amount and differentiation of bone marrow, the three BBCPs groups differed considerably; BBCP1 sites displayed the most bone marrow and the most advanced differentiated bone marrow. This appeared similar to the bone marrow found in the defect adjacent normal bony sites.

#### Histomorphometry

**Fig. 11:**
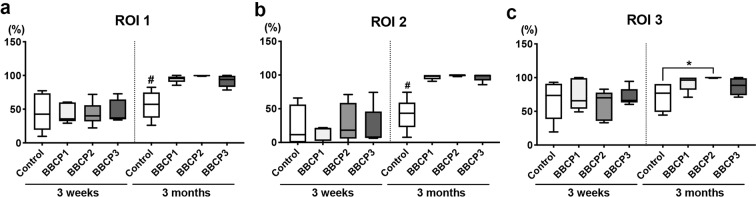
The percentage of horizontal defect closure with bone at 3 weeks and 3 months post surgery in **a** whole defect (ROI 1), **b** middle area of the defect (ROI 2), and **c** lateral area of the defect (ROI 3). *denotes significant difference between the groups (*p* < 0.05). ^#^denotes significantly lower than any other modalities (*p* < 0.05)

**Fig. 12:**
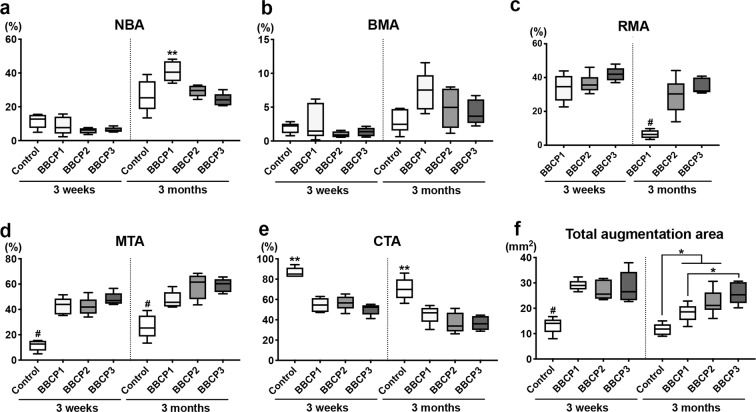
Histomorphometry for area parameter analysis at 3 weeks and 3 months post surgery in the whole defect (ROI 1); **a** new bone area (NBA, % to total augmentation area), **b** bone marrow area (BMA, % to total augmentation area), **c** residual material area (RMA, % to total augmentation area), **d** mineralized tissue area (MTA, NBA + RMA; % to total augmentation area), **e** connective tissue area (CTA, % to total augmentation area), and **f** total augmentation area (mm^2^). *denotes significant difference between the groups (*p* < 0.05). **denotes significantly higher than any other modalities (*p* < 0.05). ^#^denotes significantly lower than any other modalities (*p* < 0.05)

**Fig. 13:**
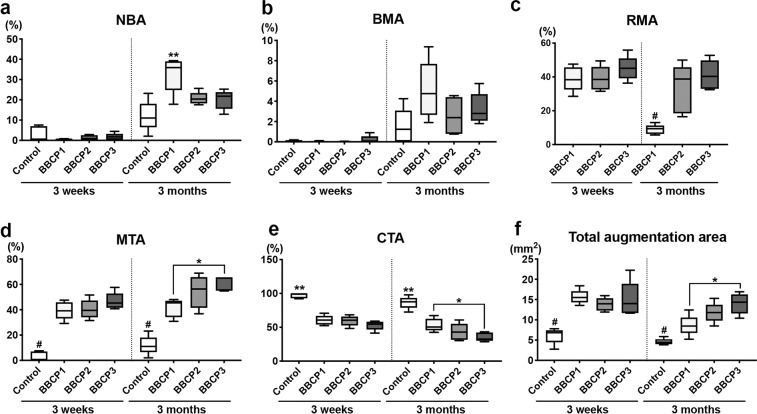
Histomorphometry for area parameter analysis at 3 weeks and 3 months post surgery in the middle area of the defects (ROI 2); **a** new bone area (NBA, % to total augmentation area), **b** bone marrow area (BMA, % to total augmentation area), **c** residual material area (RMA, % to total augmentation area), **d** mineralized tissue area (MTA, NBA + RMA; % to total augmentation area), **e** connective tissue area (CTA, % to total augmentation area), and **f** total augmentation area (mm^2^). *denotes significant difference between the groups (*p* < 0.05). **denotes significantly higher than any other modalities (*p* < 0.05). ^#^denotes significantly lower than any other modalities (*p* < 0.05)

**Fig. 14:**
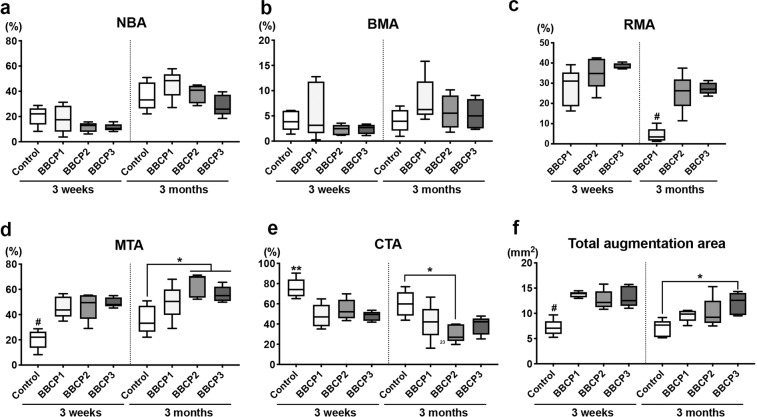
Histomorphometry for area parameter analysis at 3 weeks and 3 months post surgery in the lateral area of the defects (ROI 3); **a** new bone area (NBA, % to total augmentation area), **b** bone marrow area (BMA, % to total augmentation area), **c** residual material area (RMA, % to total augmentation area), **d** mineralized tissue area (MTA, NBA + RMA; % to total augmentation area), **e** connective tissue area (CTA, % to total augmentation area), and **f** total augmentation area (mm^2^). *denotes significant difference between the groups (*p* < 0.05). **denotes significantly higher than any other modalities (*p* < 0.05). ^#^denotes significantly lower than any other modalities (*p* < 0.05)

The percentage of horizontal defect closure with new bone was not significantly different between any groups in the three different ROIs at 3 weeks (Fig. 11). All tested BBCPs resulted in greater defect closure, when compared to the control group in ROI 1 and ROI 2 at 3 months, and only BBCP2 yielded greater defect closure than the control in ROI 3. BBCP1 group showed significantly higher NBA when compared to the other three groups at 3 months in ROI 1 and ROI 2 (Figs. 12a and 13a). Furthermore, there was significantly less residual BBCP1 compared to the other two BBCPs at 3 months in all 3 ROIs (Figs. 12c, 13c, and 14c). All BBCPs showed higher percentages of MTA and lower CTA when compared to the control group in all ROIs both at 3 weeks and 3 months (Figs. 12d, e, 13d, e, and 14d, e). The relative percentages of area parameters (Figs. 12–14) were related to the total augmentation area (Figs. 12f, 13f, and 14f). The absolute values of area parameter analyses (mm^2^) were not shown, as the tendencies of the results were similar to those of the relative percentage.

## Discussion

This study aimed to test the performance of BBCP granules comprising HA and α-TCP with respect to space maintenance, degradation, and new bone formation. To this end, three different particulate BBCP prototypes were tested in critical size defects of rabbits. All BBCPs used in this study were biocompatible and osteoconductive. The bone graft substitute with the lowest HA content (3% HA; BBCP1) yielded the highest rate of material degradation concomitant with the highest rate of new bone formation. Thus, it has been confirmed that the degradation kinetics of particulated BBCP and the formation of new bone may be modified by alternating the ratio between epitactically coated HA and α-TCP.

Rabbits are small and skeletally mature animals, reported as a suitable model for a variety of research topics. However, the difference in bone formation between the groups might be also attributed to the favorable healing capacity of the animals. Nonetheless, no single animal model might be considered ideal to answer the purposes within a specific field of research. The micro-CT and histological findings in this study indicated that these limitations may be overcame by the use of critical size defects.

Degradation of all three BBCPs in this study was observed from 3 weeks to 3 months of healing. Given that degradation kinetics of bone substitute materials are essential for their osteoconductive properties [16], the amount of epitactically coated HA in BBCP has been identified as a critical parameter. The limiting effect of HA on BBCP degradation was not unexpected, since HA is known as an inert, the least soluble calcium phosphates. The material degradation clearly depended on the HA/α-TCP ratio, with BBCP3 (23% HA) showing highest volumes of residual material and total mineralized volume. Residual volumes of 20% HA/β-TCP at 2-week healing period [19] and of 10% HA/β-TCP at 3 months [20] were not different as compared to this study. Thus, in vivo data indicate rather similar outcome regardless of different behavior of BBCPs composed of epitactically coated HA on an α-TCP core and BCPs made of HA and β-TCP. Despite the differences in degradation kinetics, the volume maintenance capacity of all three BBCPs up to 3 months were found very similar, i.e., all three HA/α-TCP provided sufficient space between the dura and the periosteum to be filled with bone. However, a conclusive evaluation of space maintenance of the herein tested biomaterials needs to be assessed in a 6-month follow-up study.

Another aspect to be considered within the context of this study is that the macrostructure of biphasic calcium phosphate as the HA/α-TCP in block form showed no significant degradation [21, 22, 24]. Unfortunately, the ratio of HA/α-TCP of the tested blocks as well as microporosity of the material has not been reported. Therefore, it is not possible to deduce if the observed differences in degree of degradation and new bone formation in case of BBCP were caused by the use of granules and their secondary macroporosity, due to the intergranular space formation after application [25, 26].

Total porosity as well as pore size, pore structure, and interconnectivity have significant impact on the biological behavior of calcium phosphate-based bone substitute materials (for review see [27, 28]). Infiltration of blood cells and presence of cells at 3 weeks inside the large and small pores of the BBCP1 granules indicated a highly interconnected pore system with an efficient fluid and cell uptake throughout the whole granule. The pore system in the other two BBCPs restricted cell/tissue uptake and ingrowth to the few large pores inside the granules. In this case bone formation was mainly restricted to the surface of the granules. It is possible that besides the known pores necessary for vessel ingrowth [29], the presence of cell accessible pores also determines the degradation of bone substitute materials and thus, the amount of newly formed bone.

Macrophages, being the precursor of multinucleated cells, have been identified as key players in biomaterial induced bone formation [30] and play an essential role in degradation of biomaterials [31]. As the layers of multinucleated cells surrounded the BBCP granules, they may be also considered the main driver for the degradation of BBCP granules based on α-TCP. In all tested sites, small concavities observed in micro-CT and histologically pointed toward a specific pattern of intrinsic osteoinduction [32–35]. It has been suggested that the intrinsic osteoinduction happens first on the surface of pores in the core of the material and then spread to the periphery [12, 25], corresponding to the BBCP1 findings most pronounced in the central area of the defects. Multinucleated giant cells are considered to play an essential role in this phenomenon [36–38]. The intrinsic osteoinduction is not caused by an accumulation, but a local reduction of calcium and/or phosphate ion concentrations through apatite formation. The exact mechanism is, however, still unclear [39]. Albeit, cell-independent dissolution of the material cannot be excluded.

To investigate the cell-independent dissolution of the overall dissolution rate, BBCP might be also immersed into PBS for 3 months. Lowering pH value can increase the α-TCP dissolution, with pH = 7 being threshold for Ca precipitation. Actually, calcium phosphates spontaneously precipitate in the cell culture medium in dependence of time [40]. The assessment of α/β-TCP solubility in vitro may not always represent in vivo conditions, characterized by a buffering capacity and the inhibition of the tissue mineralization by the expense of energy. A future, comprehensive in vitro study shall thus confirm the cell-independent dissolution properties of BBCPs.

Mature bone marrow including the presence of well-developed osteons and interstitial lamellae in particular in the periphery of the defect indicates an advanced stage of bone modeling and remodeling in case of BBCP1 samples. Similar findings were reported for different HA/β-TCP ratios, with higher fraction of bone marrow in the 10% HA compared to the 60% HA [20]. Consequently, the ratio between differently soluble calcium phosphates seems to be an efficient measure to fine-tune space maintenance properties and functional bone formation controlled via the kinetics of BCP and BBCP degradation. Nonetheless, the animal models used in biomaterial research do not exactly mimic the bone biology of humans. Further studies shell thus include the comparison of BBCPs with an HA content comparable to the biomaterials generally used in the clinics.

## Conclusions

Within the limitations of this study, all BBCP showed good biocompatibility and osteoconduction leading to the healing of critical size defects. The degradation kinetic of BBCPs was inversely related to the level of HA coating. Consequently, the maturity of newly formed bone was most advanced in 3%:97% HA:α-TCP.
